# The HPCDP Journal: celebrating a decade of impact

**DOI:** 10.24095/hpcdp.45.3.01

**Published:** 2025-03

**Authors:** The HPCDP Journal Team

**Affiliations:** 1 Public Health Agency of Canada, Ottawa, Ontario, Canada

## Introduction

*Health Promotion and Chronic Disease Prevention in Canada *(the HPCDP Journal) is uniquely positioned to serve diverse audiences by presenting valuable contributions to the field of public health from scientists within and outside of government. 

The HPCDP Journal marking its 10th anniversary under the current name is an opportune time to reflect on how far the journal, the editorial team and the community of contributors have come. 

Over the past decade, the journal’s focus on key public health priorities has sharpened, with rapid responses to unprecedented health challenges and significant strides in promoting population health intervention research. In this editorial, the HPCDP Journal Team highlights some of the most transformative milestones and publications, including the expanded focus on chronic disease and risk factor surveillance, the deepened commitment to population health intervention research and the rapid pivot at the start of the COVID-19 pandemic to address its far-reaching impact on chronic disease and health equity in Canada. 

## Expanding our focus on chronic disease and risk factor surveillance

The HPCDP Journal has been instrumental in advancing chronic disease and risk factor surveillance in Canada by publishing research articles that address numerous topics— childhood overweight and obesity trends,^1^ the environmental factors associated with autism spectrum disorder^2^ and analyses of the prevalence and patterns of multimorbidity and their associated determinants,^3^ among many others. The journal also featured articles about the economic burden of chronic diseases, for example, diabetes.^4^ Several of the papers shone a light on the progress made in understanding and measuring the complex interplay between movement behaviours and health, recognizing that physical activity, sedentary behaviour and sleep cannot be considered in isolation given their co-dependence.^5^ Important scientific contributions that advance our understanding of how to conceptualize and measure positive mental health^6^ have also been published. 

## Strengthening our focus on population health intervention research

A foundational development of the past decade has been the journal’s concerted focus on population health intervention research, a crucial area for addressing complex public health issues. This shift aligns with the principles of the “Ottawa Statement from the Sparking Solutions Summit on Population Health Intervention Research.”^7^ We are proud to have joined other leading journals in signing this statement, underscoring the commitment to advancing research that goes beyond describing problems to actively explore solutions that can make a measurable difference in the health of people in Canada. 

The Ottawa Statement also emphasized the importance of research that examines the wider social, environmental and policy-related determinants of health, rather than only addressing individual health behaviours.^7^

Over the past decade, the journal has published numerous manuscripts, theme issues and commentaries that reflect this commitment to supporting research on the impacts of interventions, the contexts in which they work best and who they benefit the most. For example, we have published research on the association of the built environment with different types of physical activities in adults^8^ and the role of food policies in shaping dietary behaviours.^9^ These studies represent the kind of transformative research that the Ottawa Statement^7^ advocates. They exemplify how population health intervention research can offer solutions to Canada’s most pressing public health challenges. 

Thematic issues have also provided actionable evidence to inform practices, programs and policies aimed at addressing major chronic conditions and risk factors—from substance use to food environments^10^ to climate change and health,^11^ and the national opioid crisis.^12^,^13^ We extend our sincere gratitude to all Guest Editors of the theme issues for their invaluable contributions. While most Guest Editors are from academia, the editorial process for a recent series on the unregulated drug toxicity crisis in Canada was enriched by the insights of and engagement with individuals with lived or living experience.^14^ This marked a first for the journal, and an experience we intend to repeat. 

Evidence reviews have emerged as a key article type in the HPCDP Journal, providing critical insights to inform policy, program design and practice. Several of these reviews, particularly those focused on public health interventions, have garnered significant recognition. For example, an overview of reviews on social media interventions to promote health equity^15^ stands out as highly cited. The journal has also featured articles showcasing methodological innovations for measuring the impacts of interventions. Notable among these was a paper on the development of indicators to evaluate the effectiveness and outcomes of age-friendly communities in Canada,^16^ providing a robust framework for assessing progress and informing future action. 

To further bridge the gap between science and policy, the HPCDP Journal also introduced a new article type in 2015, namely evidence-informed policy briefs. These articles summarize high-quality, relevant and up-to-date research-based evidence on known benefits and harms of interventions. These briefs also suggest options, indicate the costs and barriers to policy implementation and suggest strategies to address these barriers. However, only four such policy briefs have been published to date, underscoring the ongoing challenges in knowledge translation within the public health research community and emphasizing the need to expand the evidence base through applied research studies in public health in Canada. 

## Responding to the COVID-19 pandemic: an unprecedented challenge

While the journal’s focus on intervention research shifted gradually and strategically for most of the last decade, the COVID-19 pandemic presented an abrupt and all-encompassing challenge that necessitated immediate action. We promptly pivoted to address the broader health impacts of COVID-19, issuing a call for papers for an online-first publication model. The rapid response from the research community, in Canada and internationally, was remarkable. This response underscored the urgency of understanding the complex effects of the pandemic on population health. The HPCDP Journal has received 213 manuscripts on COVID-19 since March 2020, and so far 59 have been published. 

The pandemic illuminated the interconnectedness of infectious diseases, chronic conditions and health inequities among people in Canada. COVID-19 affected not only the people who were infected but also individuals with pre-existing chronic conditions who faced disruptions in care and heightened risk due to strained health care systems. The pandemic also underscored the need for equity in the approach to health promotion and chronic disease prevention. COVID-19 laid bare the vulnerabilities of certain population groups—Indigenous communities, racialized groups and individuals with low income—who experienced higher rates of infection, poorer outcomes and greater barriers to health care.^17^X 

The HPCDP Journal responded by publishing the results of studies that analyzed, in different population groups, the indirect effects of the pandemic on health behaviours and chronic disease outcomes, including on mental health outcomes.^18^

The long-term impact of the COVID-19 pandemic on health extends beyond the immediate crisis, including the effects of post-COVID-19 condition,^19^ with lasting consequences for chronic disease management and overall well-being. The disruptions in care, combined with changes in health behaviours and increased mental health challenges, have underscored the importance of long-term studies to fully understand these effects. 

As we continue to address these challenges, effective dissemination of scientific findings will be critical to shaping equitable, evidence-based strategies that promote resilience and health equity across all communities. 

## Looking forward: a vision for the next decade

Looking ahead, we recognize the importance of continuing to publish research findings that are based on, and that foster, cross-disciplinary and cross-sectoral collaborations. The challenges we face in chronic disease prevention and health promotion cannot be addressed by the public health sector alone; they require partnerships across education, housing, transportation and social services to create environments that support healthy behaviours and reduce health disparities. Our special issue on social prescribing, published in 2024, contributes to this conversation,^20^,^21^ as will the theme issue on natural experiments and built environments scheduled for release later this year. 

In this context, the HPCDP Journal also welcomes contributions in emerging fields relevant to health promotion and chronic disease prevention, such as digital health and health technology, implementation science, the commercial determinants of health, big data and predictive analytics, as well as the intersections of infectious and chronic diseases through a One Health approach. These areas reflect the evolving landscape of public health and highlight the importance of innovative and integrative approaches to improving population health outcomes. 

We are dedicated to strengthening the journal’s role as a platform for research that places equity at its core. The pandemic exposed glaring health disparities and reinforced the critical need to address the social determinants of health. In the years ahead, we aim to publish research that goes beyond documenting these inequities to testing and evaluating interventions designed to reduce them. By prioritizing equity-driven research, we aspire to contribute to a future where everyone in Canada has the opportunity to attain optimal health and well-being. 

The HPCDP Journal will also continue to publish vital research that leverages pan-Canadian surveillance systems, advancing our understanding of risk and protective factors and contributing to chronic disease prevention efforts across the country. 

Finally, we recognize the importance of embracing innovation in our publishing practices to remain responsive to the evolving landscape of scientific communication. Advances in technology are transforming how research is conducted, shared and accessed, and we are committed to ensuring that *Health Promotion and Chronic Disease Prevention in Canada* stays at the forefront of these changes. As an open-access journal that publishes original research articles, we aim to explore practices that accelerate the dissemination of research findings and to adopt new formats that enhance engagement with published work. By continually evolving our practices, we strive to better meet the needs of readers and contributors while maximizing the impact of the research we publish. 
